# Synthesis of water-soluble surfactants using catalysed condensation polymerisation in green reaction media†

**DOI:** 10.1039/d1py00415h

**Published:** 2021-04-26

**Authors:** Amy R. Goddard, Edward A. Apebende, Joachim C. Lentz, Kim Carmichael, Vincenzo Taresco, Derek J. Irvine, Steven M. Howdle

**Affiliations:** Croda Europe Ltd, Foundry Lane Ditton Widnes WA8 8UB UK; University of Nottingham, School of Chemistry, University Park Nottingham NG7 2RD UK steve.howdle@nottingham.ac.uk; Centre for Additive Manufacturing, Faculty of Engineering, University of Nottingham Nottingham NG7 2RD UK

## Abstract

Sustainable and biobased surfactants are required for a wide range of everyday applications. Key drivers are cost, activity and efficiency of production. Polycondensation is an excellent route to build surfactant chains from bio-sourced monomers, but this typically requires high processing temperatures (≥200 °C) to remove the condensate and to lower viscosity of the polymer melt. In addition, high temperatures also increase the degree of branching and cause discolouration through the degradation of sensitive co-initiators and monomers. Here we report the synthesis of novel surface-active polymers from temperature sensitive renewable building blocks such as dicarboxylic acids, polyols (d-sorbitol) and fatty acids. We demonstrate that the products have the potential to be key components in renewable surfactant design, but only if the syntheses are optimised to ensure linear chains with hydrophilic character. The choice of catalyst is key to this control and we have assessed three different approaches. Additionally, we also demonstrate that use of supercritical carbon dioxide (scCO_2_) can dramatically improve conversion by reducing reaction viscosity, lowering reaction temperature, and driving condensate removal. We also evaluate the performance of the new biobased surfactants, focussing upon surface tension, and critical micelle concentration.

## Abstract 2

Synthesis and characterisation of novel linear surface-active polymers from temperature sensitive renewable building blocks using an inexpensive catalyst and clean scCO2 as reaction medium.

## Graphical abstract

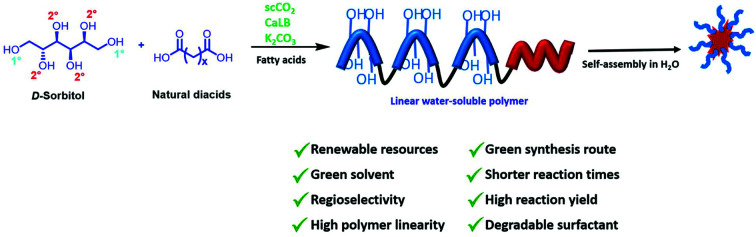

## Introduction

Successful synthesis of linear polyol-polyesters can lead to development of new sustainable surfactants^1–5^ and there is particular focus on the use of carbohydrates as a renewable resource for polymer design,^3,6,7^ because they bring a rich variety of structures with abundant stereochemical diversity.^8^ Carbohydrates such as glucose, glycerol and d-sorbitol contain multiple hydroxyl groups and are emerging as alternative hydrophilic moieties in surfactants.^1,9^ These materials could also offer a renewable alternative to other well-established hydrophilic polymers and macromonomers such as poly(ethylene glycol) (PEG), where there are increased concerns regarding toxicity.^10^ Though carbohydrates are viewed as more acceptable, only a small number are currently competitive on price, quality and availability compared to the standard petroleum derived materials.^11^ An additional problem is that polyol monomers are often thermally unstable and susceptible to side reactions such as dehydration and decarboxylation which are deleterious, leading to lowered hydrophilicity and colouration.^12^

When employing renewable hydrophiles in surfactant design, the water solubility of the compound is essential and multiple building blocks are therefore used when modifying polymer properties and tailoring end use.^11^ The number of building-blocks in commercial polyglucosides and polyglycerol is generally between 2–10 repeat units, which increases the number of free hydroxyl groups along the oligomer/polymer backbone.^13^ This number is dependent on the catalyst system employed and it is well known that there is decreased selectivity observed for acid/base catalysed homopolymerisations at high MWs.^14^ This limits the number of repeat units, as undesirable branched and cyclic side-products are formed at higher MWs, reducing the hydrophilicity of the end product.^13,15^ Furthermore, when conducting polymerisations using polyols, viscosity build and higher temperatures lead to darkening in colour and this often renders products unsuitable for personal care applications.^14,15^

To overcome concerns regarding control of molecular weight and side-product formation, the linkage of polyols using dicarboxylic acids has been reported in surfactant design, an example being the synthesis of polyglycerol diacid esters.^1,5,16^ These are then esterified with fatty acids to introduce a hydrophobic moiety, and have been commercialised under the brand NatraGem™.^17^

Condensation polymerisations conducted using dicarboxylic acids and polyols, such as glycerol and d-sorbitol, have been widely performed in the bulk, and thus are solvent-free systems.^18,19^ From a sustainability perspective this is attractive as it removes the need to use volatile organic compound (VOC) solvents. The reaction is driven forward by the removal of the condensation by-product using heat, gas sparging and also additionally can require high energy vacuum.^20,21^ However to get high conversion, the syntheses can also require high temperatures and this introduces problems with respect to hyperbranching and cross-linking.^2,22^

The use of an organic acid catalyst such as *para*-toluene sulfonic acid is well recognised in the high temperature synthesis of sorbitan ester surfactants and also highly branched polyesters. But, such organic acid catalysts area also employed in the dehydration of d-sorbitol to sorbitan and isosorbide in solvent-free systems.^4^ This dehydration is undesirable for creating surfactants as it reduces the number of hydroxyl groups in sorbitol, consequently lowering the hydrophilicity (and water solubility) of the resulting polyol.^3^ Elaborate and tedious protection-deprotection steps are often reported for linear hydrophilic polyesters, making the process too complicated for industrial application.^12^

A high degree of selectivity towards the synthesis of linear polyol-polyesters has also been reported by exploiting Novozym 435 (immobilised *Candida antarctica* Lipase B).^1,4,18^ Selective derivatisation of the primary hydroxyl groups has been identified when employing compounds like d-sorbitol^23–26^ a feature which could prove very valuable in the design of renewable hydrophiles. Nonetheless, high costs associated with enzymes hinder their widespread industrial use, and this is an important factor to consider when synthesising surfactants for commercial applications.^27,28^ Our aim in this work is to look at alternative catalysts that are already established and to compare performance directly with the high degree of control that would be expected from the use of Novozym 435.

Base catalysis has also shown success in the synthesis of water-soluble polyol polymers.^17,29^ Kiely *et al.* synthesised copolymers using dimethyl galactarate and alkylene diamines of varying chain lengths (C2–C12).^29^ Polyamides synthesised using C2 and C4 alkylene diamines, yielded oligomers with *M*_n_ ≤ 1200 Da, while the MWs were typically ∼2300 Da when using chain lengths ≥C6.

A degree of selectivity has also been displayed in the homopolymerisation of glycerol (forming linear polyglycerol oligoesters) using mineral alkali catalysts such as the hydroxides and carbonates of sodium and potassium. In particular, in previous patented examples, K_2_CO_3_ has been employed to link polyglycerol oligoesters (containing 3 to 20 glycerol units) using dicarboxylic acids (ranging from C4 to C22) in solvent-less conditions.^17,30^ The reactions were normally conducted at temperatures between 110–200 °C in order to facilitate the removal of the condensate. However, the ability to use lower temperatures would minimise branching and discoloration. The linkage of polyglycerol oligoesters at the primary hydroxyl position ensured the products were water soluble, as free secondary hydroxyl groups were available along the oligomer/polymer backbone. This is important for their end use as oil-in-water emulsifiers, sold to the personal care and home care industries. As d-sorbitol is more sterically hindered than glycerol, a higher degree of chemo-selectivity would be predicted when using this building block. Accordingly, carbonate catalysts could be used for the synthesis of linear d-sorbitol based polyesters, with the advantages of being inexpensive and well established in commercial processes.^17^

**Fig. 1:**
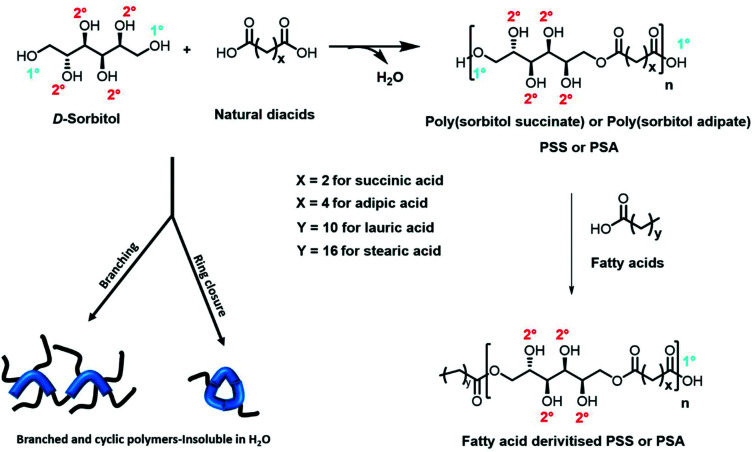
Condensation polymerisation of diacids with d-sorbitol, followed by end group functionalisation using fatty acids. Note the reaction at the primary hydroxyl groups (1°) is essential to ensure the secondary hydroxyls (2°) are free for hydrogen bonding along the polymer backbone in order to deliver hydrophilicity. Reactions catalysed by *p*TSA are non-selective and result in branched and cyclic polymers that are insoluble in water and highly coloured.

In this paper, we show for the first time the production of a library of linear polyesters derived from the model polyol sorbitol and a small selection of diacids combining K_2_CO_3_ as an inexpensive and readily available catalyst and scCO_2_ as the reaction medium to enable mild reaction conditions. In addition, the purge of the by-product condensate was easily and effectively afforded by quick scCO_2_ extraction, which increased reaction rate and conversion when compared to the bulk “solvent-less” conditions. *p*TSA was used as negative control showing the production of highly branched polymers while Novozym 435 was adopted as positive control *i.e.*, as benchmark comparison for linear functionalised polyesters. Finally, in order to tune the amphiphilic balance of the resulting hydroxylated polyester backbones we developed an end group derivatisation strategy, and the resulting surfactants were tested against commercial equivalents (Fig. 1).

## Results and discussion

### Evaluating catalysts for the synthesis of linear poly(sorbitol diacid) polyesters in bulk conditions

The use of *p*TSA, Novozym 435 and K_2_CO_3_ for the synthesis of poly(sorbitol diacid) polyesters were compared in terms of the conversions reached, molecular weights achievable, OH : COOH ratios and the visual appearance of the polymers obtained. Conversions were calculated by comparing the integrated values of monomeric adipic acid (ROCC**H2**–) to its reacted (oligomer or polymer) form (4H, m, *δ* = 2.02–2.10 to *δ* = 2.29–2.33 ppm, from **a** to **a′**) (SI, Fig. S1†). Note that the polyester end-group HOOCC***H*2**– (**a′′**) also has a shift in this region (2H, m, *δ* = 2.02–2.10). This will influence the conversion measurements determined by ^1^H-NMR making them a conservative estimate. Peaks at *δ* = 3.23–5.30 ppm (**c′**, **d′** and **e′**), correspond to d-sorbitol, though the presence of water (at *δ* = 3.30 ppm) makes integrations unreliable. In all cases, conversions are compared to values determined by acid value titration (PA%). The use of *p*TSA as catalyst yielded the highest conversion (93% at 120 °C). The product, however, was poorly soluble in water indicating a significant loss of –OH groups to undesired ring closing and crosslinking. It was also dark in colour suggesting that thermal and oxidative degradation processes influenced by the presence of contaminants and side-products could have also occurred.^9^ Mass spectrometry indicated that synthesis using *p*TSA also resulted in esterification at the secondary hydroxyl groups (ESI, Fig. S2†), leading to the undesirable deviation from linearity in the product and also causing a decrease in the hydrophilicity and hence water solubility.

Ring closed sorbitan is an indicator of dehydration and also shows that ring closure side reactions took place alongside polymerisation in the presence of *p*TSA.^3,9^ Peaks corresponding to sorbitan derivatives were detected exclusively in the product synthesised with *p*TSA. The darkening in colour would make these products undesirable for personal care applications, and their reduced solubility would make them unsuitable as hydrophilic components in surfactant design. For these reasons *p*TSA was not further investigated. On the other hand, reactions catalysed using Novozym 435 and K_2_CO_3_ were much more promising; the products were found to be soluble in aqueous environment and considerably lighter in colour. In addition, the enzyme could be washed and re-used as has been previously demonstrated.^31,32^ Potentiometric titrations showed that there are considerably more hydroxyl groups than carboxylic acid groups in the samples from the enzymatic and carbonate catalysed polymerizations (Table 1). This excess of hydroxyl groups suggests that ring closure and branching, which are a consequence of the reaction of the secondary alcohol groups of sorbitol, have been minimised. Clearly, this also explains the high aqueous solubility of these compounds. However, with both Novozym 435 and K_2_CO_3_, it can be seen that the OH : COOH ratio is slightly less than their expected (theoretical) values (see ESI Table S1†) indicating that reactions at the secondary hydroxyl groups may not have been eliminated completely. It is also possible that the titrations could be giving slightly lower hydroxyl values because acetylation of the secondary hydroxyl groups can be challenging due to steric hindrance. We can see this in the titration of pure d-sorbitol, which gave an average of 5.8 hydroxyl groups instead of the theoretical value of 6. It is also important to note that there is very likely a contribution from reactions at the secondary hydroxyl groups of sorbitol. This is something that we have investigated in the past and there is clear evidence that the enzyme does deliver a small level of esterification at the secondary alcohol moiety as measured by phosphitylation.^33,34^ However, the mass spectrometry data strongly hint that over esterification, and degradation of sorbitol was minimized when using Novozym 435 and K_2_CO_3_ (ESI, Fig. S2†) and it is surprising that K_2_CO_3_ would catalyse the polymerization with such close specificity to Novozym.

**Table 1:** Comparing synthesis of poly(sorbitol adipate) (PSA) using bio- and chemo-catalysts, synthesised at 95 °C and 120 °C under solvent free conditions for 48 hours

Entry	Catalyst	Conversion^a^ (%)	Ratio OH : COOH^b^	*M*_n_ (g mol^−1^)^b^	Appearance
1	*p*TSA	93	n/a	1600 ± 50	Sticky dark brown solid
2	N435	82	13 : 1	1100 ± 100	Yellow oil
3	K_2_CO_3_	83	20 : 1	1700 ± 100	Pale yellow oil

a Determined by H-NMR.

b Determined by potentiometric titration.

Molecular weight determinations using acid value titrations (*M*^AV^_n_) indicate that the poly(sorbitol adipate) (PSA) synthesised using K_2_CO_3_ has a higher *M*^AV^_n_ than the equivalent compound synthesised enzymatically; 1700 Da compared to 1050 Da respectively (Table 1, entries 2 and 3).

Polyol-polyesters were analysed by inverse-gated ^13^C-NMR spectrometry which allows quantification. The polyesters synthesised using K_2_CO_3_ and Novozym 435 were identified to be surprisingly similar (ESI, Fig. S3†). The major signals, labelled 1–6, were assigned to d-sorbitol that has been incorporated into the polyester backbone. Carbons from unreacted d-sorbitol have different chemical shifts (labelled 1*–6*), with end-groups identified at a slight upfield/downfield shift (labelled 1′–6′). The significant downfield shift that was identified post-derivatisation for –CH_2_O– groups suggests that d-sorbitol has been esterified with a high selectivity at the primary hydroxyl positions. This same observation was also reported by Gross *et al.*^35^ and Gustini *et al.*^26^ when using Novozym 435. What is surprising in our data is that inexpensive K_2_CO_3_ should exhibit very similar regioselectivity to Novozym 435. Thus, in our studies we determined that only K_2_CO_3_ should be further investigated as an inexpensive, safe, and green catalyst in scCO_2_ for synthesis of water soluble polyesters.

### Synthesis of linear poly(sorbitol diacid) polyesters in scCO_2_ using K_2_CO_3_ catalysis

The potential of using supercritical CO_2_ as a reaction medium and as a potential extractant to facilitate condensate removal was evaluated. A “react, extract, repeat” approach would in theory allow the system to equilibrate, followed by condensate removal by extraction, shifting the equilibrium towards longer chain lengths. The plasticising effects of scCO_2_ on the polymerisation could also be interesting, as it is well known that decreased viscosity can lead to enhanced mass transfer properties and increased rates of reaction.^20,21^

**Fig. 2:**
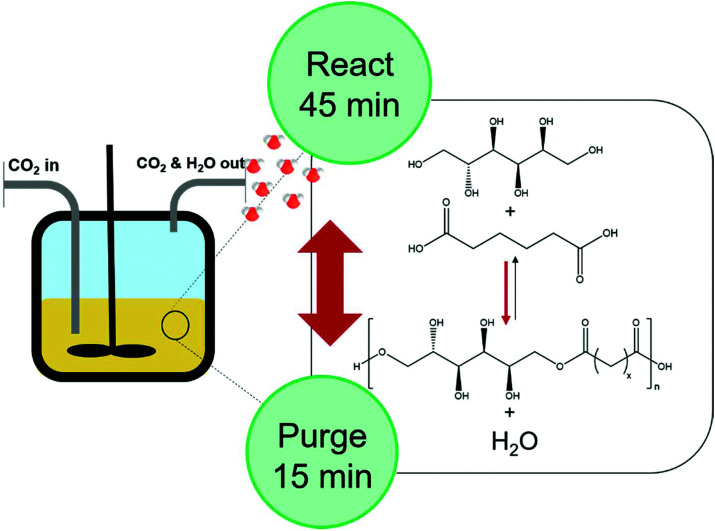
Schematic representation of the condensation polymerisation of adipic acid with d-Sorbitol in scCO_2_ in a reactor vessel. The reactor is filled with CO_2_ through an inlet while CO_2_ and H_2_O exit through the outlet on the right when the reactor is purged.

Extraction was found to be essential for the synthesis of PSA when using scCO_2_. In fact, reactions conducted in a static scCO_2_ system reached a maximum conversion of 56% with a low *M*_n_ of 550 Da (DP of 1.5) (Table 2, entries 1 and 2). Extracting periodically to remove the condensate increased the conversion to 91% and resulted in a *M*_n_ of 3300 Da (DP = 11) (Table 2, entry 6). The increased conversion and molecular mass as a result of extraction shows that the equilibrium is shifted to favour longer chain length polymers. It should also be pointed out that the insolubility of the monomers in scCO_2_ (ESI, Fig. S4†) was advantageous and allowed selective extraction of the condensate, while retaining the monomers and any low molecular weight oligomeric material inside the reactor (Fig. 2).

**Table 2:** Varying reaction conditions during synthesis of PSA in scCO_2_ using K_2_CO_3_ as catalyst

Entry	Temperature (°C)	Time (h)	Conversion^a^ (%)	*M*_n_ b (gmol^−1^)
1^c^	100	24	37	n/a
2^c^	120	48	56	550
3	120	6	62	500
4	120	12	70	800
5	120	24	81	1500
6	120	48	91	3300

a Determined by H-NMR.

b Determined by potentiometric titration.

c No extraction during reaction.

At 120 °C, a conversion of 81% was achieved after 24 hours when using scCO_2_ (Table 2, entry 5), which is equivalent to what was obtained after 48 hours in the bulk using a low pressure sparge (Table 1, entry 3). After 48 hours, a conversion of 91% was achieved when employing scCO_2_ extraction, an 8% increase compared to processing in the bulk. Whilst this increase in conversion does not appear to be substantial, it has a noteworthy impact on the molecular weight which increases from 1700 Da to 3300 Da equating to an increase in degree of polymerisation from 6 to 11. These levels of conversion were not achievable in the conventional bulk system and they can be attributed to the efficient removal of water and the higher diffusivity of the oligomers/polymer that scCO_2_ also confers. These results suggest that employing scCO_2_ during the synthesis of d-sorbitol based polyesters could effectively halve the reaction times and drive the conversion to levels that simply cannot be achieved following conventional bulk methods.

It was also found that the products synthesised with scCO_2_, but without periodic extractions, were visibly darker and showed similar discolouration to that observed during the synthesis in bulk using catalytic *p*TSA. In a static scCO_2_ system, the water formed during polymerisation remains inside the reaction vessel. Not only can this ensure the equilibrium is in an unfavourable position, but water can also react with carbon dioxide to form carbonic acid. As seen in the case of *p*TSA catalysed polymerisations, sorbitol can undergo undesired side reactions when subject to acidic conditions. The presence of carbonic acid could therefore in a similar fashion to *p*TSA drive the dehydration of sorbitol and cause undesirable side-product formation. Continuous extraction overcomes this issue as the water is swiftly removed from the reactor before these side products are able to form.

**Fig. 3:**
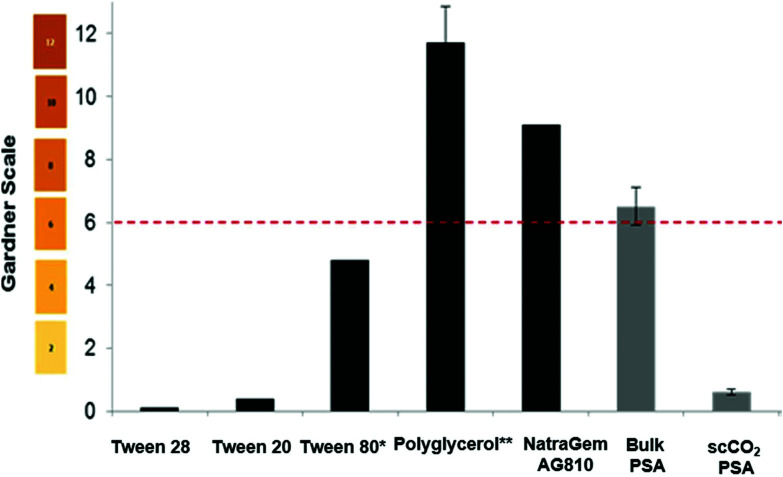
Gardner values for a range of commercial surfactants (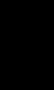) compared against poly(sorbitol adipate) synthesised in the bulk and in scCO_2_ (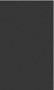), at 50% solids in aqueous solutions. The bio-based surfactant NatraSense™ AG810, polyglucose derivatised with C8–C10 fatty alcohol, has been bleached with NaOH/H_2_O_2_ post derivatisation. The Gardner colour scale is displayed for the values on the left. * At 100% solids, ** average of three samples synthesised in-house at Croda. The dashed line indicates the colour target for bio-based surfactants. Note the low colour achieved for the synthesis of poly(sorbitol adipate) in scCO_2_.

The amount of “yellowing/browning” in a sample can be measured objectively by the Gardner colour scale. This method is adopted industrially for grading the colour of resins, surfactants, oils, and fatty acids. The scale ranges from pale-yellow to a brown red in shade and is described in terms of the values 1–18. This scale was used to compare a range of commercial polyesters and surfactants to the poly(sorbitol adipate) synthesised in the bulk, and in scCO_2_ (Fig. 3). Samples were typically measured at 50 wt% in aqueous solutions. Tween™ surfactants (ethoxylated sorbitan), were inherently light in colour (Fig. 3) but polyglucose and the alkyl polyglucose derivative; NatraSense™ AG810, supplied by Croda showed the high colour values that are typical of bio-based surfactants as a result of the caramelisation, dehydration and side-product formation that occurs during their syntheses. If we compare our poly(sorbitol adipate) synthesized in the bulk to NatraSense™ AG810, a substantial improvement in colour is observed without the need for bleaching. This colour is improved even more significantly to a value of 2 when using scCO_2_ with extraction. This is a significant finding, highlighting a method of minimizing discolouration without the need for harsh post-reaction bleaching steps that often used to improve colour of surfactants for personal care products.

Polymerisations were also attempted at a lower temperature of 100 °C, but only a low conversion of 37% was achieved after 24 hours (Table 2, entry 1). The low conversion is likely to be caused by the reagents not being fully liquefied at this temperature. Although it is desirable to work at lower temperatures, this is clearly not efficient and so all further reactions were carried out at 120 °C. The “react, extract, repeat” approach in scCO_2_ was therefore also applied to synthesise poly(sorbitol succinate) at 120 °C. Results of this reaction are shown in Table S3 of the ESI.†

### Functionalising with fatty acid end groups – developing a surfactant

Sorbitol based polyesters, PSA and PSS were explored as hydrophilic components in surfactant design. The aim was to selectively end-cap the terminal primary hydroxyl groups on the polyol-polyesters *via* esterification with hydrophobic fatty acids.^1^ Simultaneously maintaining aqueous solubility and molecular weight but avoiding over- and transesterification.

Derivatisation of the PSA and PSS chains was controlled by targeting the polyester chain to end-cap feed ratios. When observed through a view cell as illustrated for PSA synthesis in (ESI Fig. S4†), the reactants were seen to be initially heterogeneous, possessing distinct fatty acid and polyester layers. With time, these phases morphed into a homogeneous emulsion as the polyol-polyester derivatives which act as surfactants were formed. This is supported by findings from Ducret *et al.*^36^ who identified increased solubility of d-sorbitol in an oleic acid phase as soon as small amounts of monomer were produced. Post reaction, the samples were washed with chloroform to remove unreacted fatty acid and the final products were found to be light amber water soluble solids. The reaction was monitored using ^1^H-NMR, though exact conversions could not be estimated as resonances from the fatty acid (**c**, 2.33 ppm) and the poly(sorbitol adipate) backbone (**a**, 2.2–2.4 ppm) overlap (ESI Fig. S5†). This overlap is also apparent for poly(sorbitol succinate) end-groups (2.22–2.43 ppm). However, the percentage of fatty acid integrated into the polyester backbone could be determined by assessing the integrals of the triplet at 0.85 ppm (**g**, terminal methyl group in lauric and stearic acid) against peaks at 1.35–1.60 ppm (**b**, **b**′, poly(sorbitol diacid) backbone protons). Results from the end-capping of the polymers are summarised in Table 3.

**Table 3:** Synthesis of end-capped poly(sorbitol diacid), using lauric acid and stearic acid

Entry	Polyester	Fatty acid	[Polyester] : [FA]^a^	Reaction time (h)	Yield^b^ (%)
1	PSA	Lauric	1 : 1.3	6	88%
2	PSA	Lauric	1 : 1.3	24	96%
3	PSA	Stearic	1 : 1.2	24	94%
4	PSS	Lauric	1 : 1.5	24	98%
5	PSS	Stearic	1 : 1.5	24	97%

a Based on *M*^AV^_n_ of polyester.

b Yield: weight of collected product/theoretical weight.

High gravimetric yields (>94%) were achieved in all cases except for entry 1 where it was found that 6 hours was too brief a time to achieve a significant amount of end-capping. Consequently, the reaction mixture was unable to form a single homogeneous phase on this timescale. Low end capping efficiency means a biphasic system persists, hampering the reaction between the hydrophobe and hydrophile. Reactions that were allowed to proceed for 24 hours were able to overcome the initial biphasic period of the reaction and form a homogeneous phase.

### Surfactant tension measurements of d-sorbitol-based polyesters: dynamic and static

**Fig. 4:**
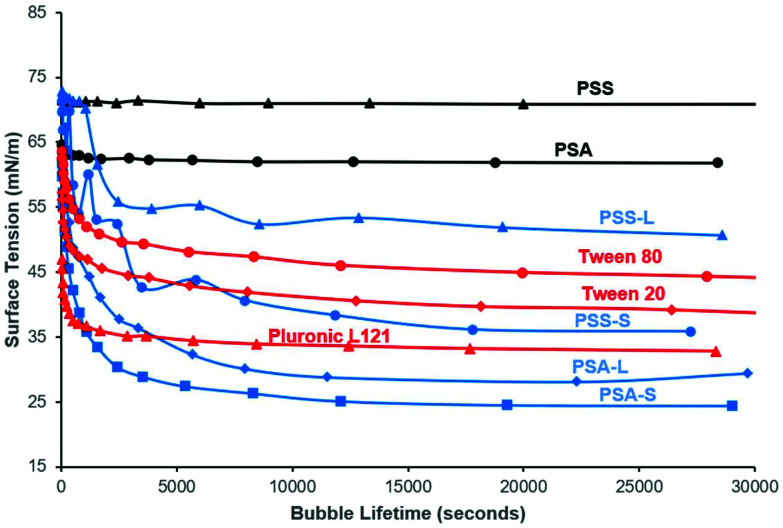
Bubble Tensiometer determination of surface tension at increasing bubble lifetimes for synthesised polyesters PSA (poly(sorbitol adipate)) and PSS (poly(sorbitol succinate)) (black curves), their stearate and laurate derivatives (blue curves) and commercial surfactants (red curves). The method shows how fast the compounds migrate to the bubble-water interface. Note the reduction in surface tension for the derivatised polyesters, and that they are competitive with PEG-based commercialised surfactants Tween™ and Pluronic™.

To confirm the surface activity of the synthesised d-sorbitol based polyesters, dynamic and static surface tension measurements were performed. Results were compared to surface tension reductions achieved using commercial Tween™ and Pluronic™ surfactants that are based on petrochemical sourced PEG (polyethylene glycol) and PPG (polypropylene glycol). Neither the starting materials (d-sorbitol and dicarboxylic acids), nor the polyesters PSA and PSS reduced the surface tension of water below 60 mN m^−1^, when measured dynamically (Fig. 4). However, all the end-capped polyesters reduced the surface tension significantly as has been shown previously.^15^ The end-capped PSAs reduced the surface tension of water to ∼30 mN m^−1^, with the compounds quickly migrating to the surface interface, identified by a rapid drop in surface tension (bubble was present only for short period of time; lifetime ∼5000 seconds). PSS based compounds appeared to migrate to the surface at a slower rate, with a maximum reduction in surface tension being ∼50 mN m^−1^ for the laurate and ∼40 mN m^−1^ for the stearate derivative (where a reduction in surface tension was apparent at bubble lifetimes up to ∼18 000 seconds). These values are comparable to the Tween™ surfactants which reduced the surface tension to ∼40–50 mN m^−1^, while Pluronic™ 121 reduced the surfaced tension to ∼35 mN m^−1^ (Fig. 4).^4^ The Pluronic™ surfactants quickly migrated to the interface causing a sharp drop in surface tension, at low water-bubble lifetimes (<2500 seconds). The surface tension therefore quickly reaches a plateau as the equilibrium surface tension is achieved. This drop in surface tension was found to be slower for the Tween™ surfactants and a decrease in surface tension was observed at bubble lifetimes as low as ∼480 seconds. The rate of reduction before reaching a plateau is dependent on the concentration and the structure of the compound evaluated. The surface tension is likely to plateau within a shorter timeframe with increased concentrations (>1 wt%), as identified previously for cationic and non-ionic surfactant systems.^30^

**Fig. 5:**
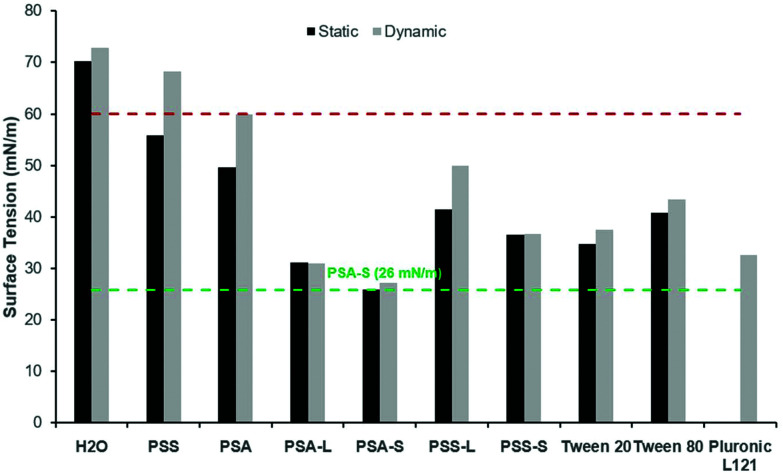
Static (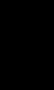) (0.5 wt%) and dynamic (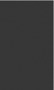) (1 wt%) surface tension (*σ*) measurements of synthesised polyesters compared to a range of commercialised surfactants. Compounds reducing *σ* ≤ 60 mN m^−1^ (red dashed line) are classed as surfactants. The lowest surface tension measurement obtained is 26 mN m^−1^ for PSA-S, highlighted (green dashed line) to aid visual comparison. PSS: poly(sorbitol succinate), PSA: poly(sorbitol adipate), L: laurate, S: stearate, Tween™ 20: PEG_20/80_-sorbitan laurate, Tween™ 80: PEG_20_-sorbitan oleate and Pluronic™ L121: PEG_5_-PPG_60_-PEG_5_.

In all cases the static (equilibrium) surface tension measurements were lower than the equivalent measurements using the dynamic tensiometer (Fig. 5). This would be expected as the aqueous surfactant system will be in equilibrium using the static test method. PSA and PSS were measured and found to reduce the surface tension of water slightly to 50 mN m^−1^ and 56 mN m^−1^ respectively. This apparent surface activity is likely caused by the difference between the hydrophilic d-sorbitol repeat units and the hydrophobic hydrocarbon backbone on the dicarboxylic acid. Thus, as the carbon chain is increased from C4 to C6 (for succinic and adipic acid, respectively) a greater reduction in the surface tension is identified. When they are end-capped with fatty acids this alters the amphiphilic balance, and a considerably greater reduction in surface tension is observed (Fig. 5). Derivatisation of PSS with lauric and stearic acids leads to surface tension measurements of 41 mN m^−1^ and 36 mN m^−1^ respectively, decreasing as the fatty acid chain length increased.

As identified using the dynamic surface tension test, the greatest reduction in surface tension was observed for PSA derivatives at 26–31 mN m^−1^. In this case, the reduction is more than doubled compared to the underivatised polyester. These results are very competitive to commercial petrochemical-based surfactants (Tween™ and Pluronic™) and are lower than d-sorbitol esters previously reported (38–46 mN m^−1^).^17^ The results are also closely matched with the commercial bio-based surfactant NatraGem™ E145 (polyglycerol succinate laurate), at 29 mN m^−1^. This is expected as their structures are relatively similar; both based on sugar polyols, dicarboxylic acids and oleochemical building blocks.

### Surfactant aggregation and micellisation

#### Critical micelle concentration (CMC)

CMC, the concentration at which the surfactant molecules self-organize in solution into micelles can be detected from surface tension *vs.* concentration graphs at the point where the surface tension reaches a plateau or remains fairly constant.^37^ With the synthesized polyesters, the surface tension of PSA-S was identified to drop to ∼29 mN m^−1^, before plateauing at this value allowing the CMC value to be determined at 0.003 wt% (ESI, Fig. S6†). For the non-capped polyesters (PSA and PSS), no drop or plateau was identified at concentrations ≤1 wt% (Table 4, entries 1 and 2). This is in line with expectation as they are highly hydrophilic, easily dissolve in the water and do not significantly alter the surface tension.

**Table 4:** CMC of the synthesised polyester surfactants determined by automated surface tension measurements using a Wilhelmy plate at concentrations from 0.1–10 000 mg L^−1^

Entry	Product	*M*_n_ (g mol^−1^)^a^	CMC		
			mg L^−1^	wt%	μM
1	PSA	1700	>10 000	>1%	>5900
2	PSS	5900	>10 000	>1%	>1700
3	PSA-L	1900	220	0.022%	120
4	PSA-S	2000	30	0.003%	15
5	PSS-L	6100	5080	0.508%	830
6	PSS-S	6200	2040	0.204%	330
7	Tween™ 28^b^	3900	2650	0.265%	680
8	Tween™ 80^c^	1300 Da	1.2	0.002%	—
9	Tween™ 20^c^	1200 Da	5.9	0.007%	—

a Calculated from *M*^AV^_n_ of poly(sorbitol diacid) + fatty acid.

b PEG_80_ sorbitan laurate.

c Reported CMC values.^4^

By contrast, the derivatised polyesters containing the smallest hydrophilic segments; PSA-L and PSA-S (Table 4, entries 3 and 4), displayed the lowest CMC values. PSS derivatised products (Table 4, entries 5 and 6) displayed higher CMC values than their PSA analogues. The difference in CMC values expressed in mg L^−1^ is reduced when expressed in the molar concentration, μM (accounting for the higher MW of the PSS based surfactants). In both cases the stearate derivatives displayed a lower CMC compared to their laurate counterparts (Table 4, entries 4 and 6 compared to entries 3 and 5).

This can be explained by the presence of a larger hydrophobic segment, as reported for similar systems elsewhere. Moreover, the CMC values determined for the PSA derivatives and PSS-S are much lower than the values observed for Tween™ 28 (Table 4, entry 7). On the other hand, Tween™ 80 and Tween™ 20 display low CMC values, at 0.002 and 0.007 wt% respectively (Table 4, entries 8 and 9). Of the synthesized surfactants, PSA-S shows a comparable CMC value of 0.003 wt% (Table 4, entry 4). When comparing molar concentrations, the CMC of PSA-S is slightly higher (at 15 μM) compared to Tween™ 80 and Tween™ 20 at 1.2 and 5.9 μM respectively (Table 4, entries 8 and 9).

### Dynamic light scattering

DLS measurements indicate that the sizes of the self-assembled structures range from 130 to 220 nm (Table 5, entries 1–4). This suggests that the self-assembly of the end-capped polyesters leads to the formation of aggregated nanostructures (micellar aggregates) since single spherical micelles made from surfactant molecules cannot be larger in radius than the length of a fully stretched out surfactant.^38^

**Table 5:** Size distribution of surfactant micelles measured by DLS, plotting size distribution by intensity. PSS and PSA represent poly(sorbitol succinate) and poly(sorbitol adipate), whereas L/S refer to the corresponding laurate and stearate derivatives. PDI gives an indication of the width of the overall distribution, assuming a single mean

Entry	Compound	Concentration (wt%)	*Z*-average (*d*, nm)	PDI
1	PSS-L	0.25%^a^	220 ± 15	0.31 ± 0.02
2	PSS-S	0.50%^a^	197 ± 9	0.34 ± 0.05
3	PSA-L	0.06%	171 ± 9	0.30 ± 0.02
4	PSA-S	0.06%	129 ± 2	0.287 ± 0.003
5	Pluronic™ L121	0.06%	169 ± 2	0.160 ± 0.008
6	Tween™ 20	0.06%	820 ± 30	0.730 ± 0.004

a Concentrations close to CMC value.

**Fig. 6:**
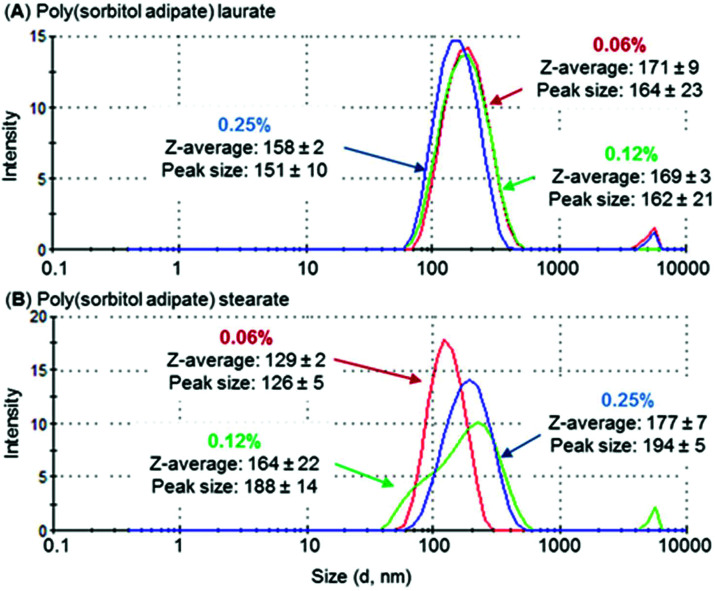
DLS measurement of (A) poly(sorbitol adipate) laurate and (B) poly(sorbitol adipate) stearate at increasing concentrations 0.06 (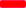), 0.12 (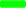) and 0.25 (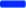) wt%, plotting size distribution (*d*, nm) by intensity. Note the increase in size for the stearate derivative, whereas the laurate polyester remains relatively constant. Peaks at higher hydrodynamic volumes (<2%) are thought to be caused by dust particles interfering with the reading.

The length of the hydrophobic (fatty acid chain) block was identified to influence aggregation size. For both PSS and PSA polyesters, a decrease in aggregate size was observed as the fatty acid chain length increased (Table 5, entries 1 and 3 compared to entries 2 and 4), with hydrodynamic volumes being ∼10–25% smaller for stearate compared to laurate derivatives. This trend was also identified for polyesters based on PEG-poly(hexanediol azelate).^39^ In this case aggregate/micelle size was identified to reduce when increasing the hydrophobic poly(hexanediol azelate) chain length. A similar behaviour was observed for PEG-PCL micelles where this was attributed to the ability of the hydrophobic core to pack tightly in crystalline regions.^40^ As PSA derivatives displayed the lowest hydrodynamic diameter, they were analysed further as a function of concentration (Fig. 6). Whilst the aggregate size of PSA-L remained relatively constant as surfactant concentration increased (between 158–171 nm), the size of PSA-S increased noticeably (from 132 nm to 180 nm). The difference in behaviour between the two end-capped PSA polyesters is most likely related to their hydrophilic : hydrophobic balance. The stearate derivatives have larger hydrophobic segments, potentially making it easier for them to assemble into elongated aggregates. This type of behaviour has been identified previously, and a rise in hydrodynamic volume has been reported to occur with time and concentration.^41^ An example is the change in aggregation size identified for sodium capped PLA oligomers.^42^ These compounds were identified to increase in diameter over a 24-hour period from 100–300 nm, suggesting the formation of larger aggregated structures with time.

### Dispersing a hydrophobic dye

**Fig. 7:**
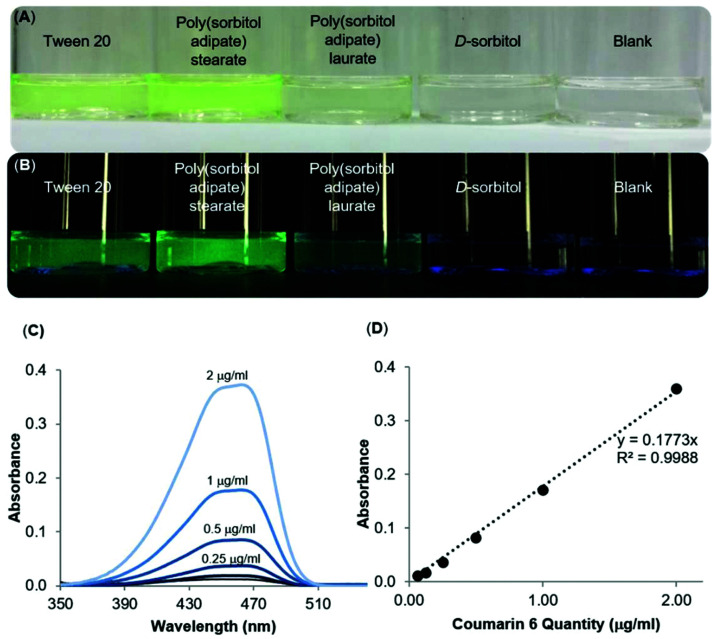
Dispersion of the highly hydrophobic fluorescent dye, coumarin 6 in aqueous solutions of synthesised polymers; poly(sorbitol adipate) stearate, poly(sorbitol adipate) laurate compared to its dispersion in commercial surfactant, Tween™ 20, a control solution with d-sorbitol and a blank solution containing only the dye viewed under (A) normal light, (B) UV light (*λ* = 365 nm, (C) UV-Vis spectra of different concentrations of coumarin 6 dye in THF:water measured from 320–520 nm and (D) a standard curve of the dye for quantification of the dispersed dye in the samples. Note the line of best fit with *R*^2^ > 0.99.

The surfactant properties of the poly(sorbitol diacid) derivatives were further probed with a fluorescent dye, coumarin 6, used as a hydrophobic drug model to encapsulate into the core and to better visualize the self-assembled aggregates. Samples were prepared by making a solution of the dye in DCM then adding the dye solution dropwise to an aqueous solution of the surfactants. The mixture was then stirred for an hour at atmospheric pressure and for 30 min under reduced pressure (75 mbar) to remove DCM. Once the DCM was evaporated, the samples were placed in an orbital shaker overnight before filtering through membrane syringe filters (0.45 μm, Millex. L.G, Millipore, USA), to exclude larger aggregates and undissolved coumarin 6. The d-sorbitol based polyesters were compared to Tween™ 20 and Pluronic™ L121, two commercially available amphiphilic surfactants used for emulsification, solubilisation and stabilisation. In particular, Tween™ 20 was used for oil-in-water systems (HLB >10) whereas Pluronic™ L121 forms water-in-oil solutions (HLB <10).^30^ Visual observations of filtered solutions of a blank sample containing no polymer and a control sample containing d-sorbitol gave a direct insight into the ability of the compounds to disperse and solubilise the dye in water (Fig. 7A and B).

**Fig. 8:**
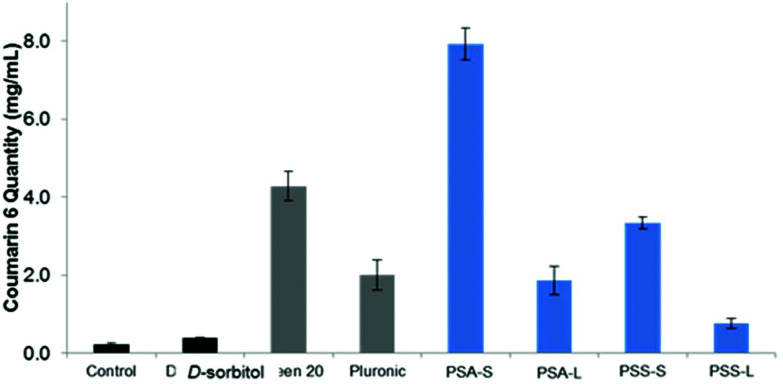
Coumarin 6 loading (μg mL^−1^) in solutions containing commercial surfactants (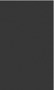, Tween™ 20 and Pluronic™ L121), synthesised surfactants (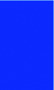, PSA/S: poly(sorbitol adipate/succinate), L: laurate, S: stearate), compared to controls (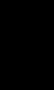, d-sorbitol and blank, containing no polymer). The polyester poly(sorbitol adipate) stearate showed the highest loading in water.

At first glance, it is clear that Tween™ 20 and PSA-S, the derivative with the longest hydrophobic segment, are able to disperse the highest amount of coumarin 6 in the aqueous phase (Fig. 7A). However, PSA-S seems to fluoresce the most when irradiated with UV light (Fig. 7B). The amount of coumarin 6 solubilised was quantified through UV-Vis analysis, by diluting small aliquots of aqueous dispersions in THF. Absorbance results at 469 nm were then quantified using a standard curve, plotted using known concentrations of the dye dissolved in a THF : water solution (Fig. 7C and D). The UV-Vis results confirmed the visual observations that the surfactants enhance the dispersion of the dye and the change in colour of the solution. Fig. 8 shows coumarin 6 dye loading in solutions containing the synthesized polyesters compared to the blank, the control and the commercial surfactants Tween™ 20 and Pluronic™ L121. As expected, d-sorbitol alone did not increase the dye loading in water. Despite this compound being fully water soluble, the lack of a hydrophobic moiety renders the compound ineffective in dispersing the dye in the aqueous solution. PSS-L, containing a relatively shorter hydrophobic segment, showed poor coumarin 6 loading (0.8 μg mL^−1^), only 2.5 times greater than the native solubility of the dye in water (0.23 μg mL^−1^). PSS-S and PSA-L on the other hand showed loadings comparable to Pluronic™ L121 and Tween™ 20 at around 2–4 μg mL^−1^ of dispersed dye. PSA-S showed the highest coumarin 6 loading at ∼8 μg mL^−1^. This was three times higher than commercial Pluronic™ L121, almost double the concentration of the commercial Tween™ 20 (at 4.28 μg mL^−1^) and around 35 times the measured native solubility of coumarin 6 in water (0.23 μg mL^−1^). These data support the hypothesis that the dye is packed differently into the core of the self-assembled aggregates based on the hydrophilic : hydrophobic ratio of the polyester alongside the size of the micelles/aggregates formed.

### Hydrolytic stability

**Fig. 9:**
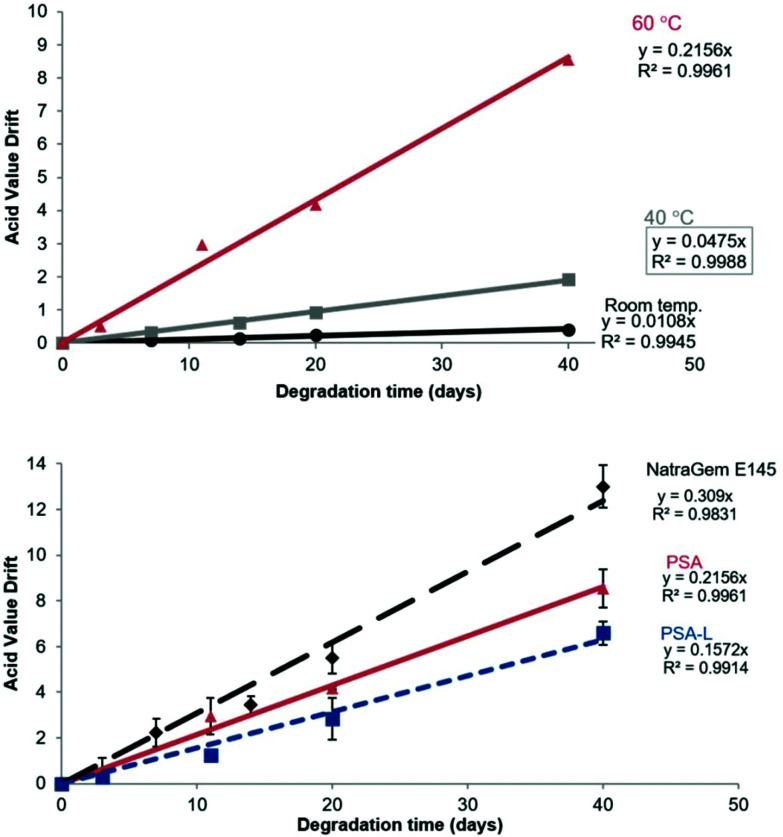
Acid value drift of (A) poly(sorbitol adipate) stored in 50 : 50 MeOH : H_2_O (v/v) at 10 g L^−1^ over 40 days at RT (●), 40 °C (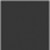) and 60 °C (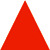) and (B) Acid Value drift comparison of; poly(sorbitol adipate (PSA, 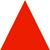), derivatised poly(sorbitol adipate) laurate (PSA-L, 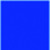) and the commercial NatraGem™ E145 (polyglycerol succinate laurate, ◆) at 60 °C. Note the reduction in acid value drift upon derivatisation.

Hydrolytic stability is an important factor to consider when making formulations for personal care applications as these often contain high levels of water.^15^ Hydrolytic stability gives an indication of how the products will react if exposed to humidity during storage, and when used on the skin. The hydrolytic stability of a product can be determined by monitoring its Acid Value (AV), indicated as the milligrams of KOH required to neutralise a gram of chemical substance, after exposing it to humidity. PSA does not readily undergo hydrolysis when stored in a 50 : 50 (v/v) methanol : water solution at RT. Effectively no drift in AV was measured at ambient temperature over 40 days. Only a small increase in AV (2 mg KOH per g) was observed at 40 °C (Fig. 9A). By extrapolating the graphs, changes to AV with time could be predicted. For the acid value to increase by 5 mg KOH per mg, PSA would need to be stored in the solution for >460 days at RT, or >100 days at 40 °C. When the temperature was increased to 60 °C the hydrolytic stability was noticeably reduced and a 5 mg KOH per mg increase in acid value was obtained after only 23 days. However, a decrease in hydrolytic degradability of PSA was identified post derivatisation with lauric acid, when measured at 60 °C (Fig. 9B).

Upon derivatisation with a fatty acid hydrophobe, the compounds were found to aggregate. This leads to a reduction in polyester-water interactions. This self-assembly could therefore explain the reduced acid value drift identified post-derivatisation. Interestingly, a greater AV drift was identified for NatraGem™ E145 (polyglycerol succinate laurate) compared to PSA and its laurate derivative. This improved stability of the d-sorbitol based polyesters compared to the glycerol-based polyesters could be caused by increased steric hindrance around the ester bonds making the hydrolysis reaction more challenging. This reduction compared to the commercial sugar-based polyesters indicates that an acceptable (even improved) level of hydrolytic degradation is occurring. However, it still shows that degradation will still occur with time ensuring an environmentally benign end of life option.

## Conclusion

We have developed an efficient and clean synthesis of d-sorbitol and diacid based polyesters by combing the use of the inexpensive K_2_CO_3_ as selective catalyst in the green medium scCO_2_. The resulting renewable polyesters were evaluated as components in surfactant design. The “react, extract, repeat” approach facilitated moderate temperatures (≤120 °C) and higher conversions allowing the removal of the condensate. This catalytic strategy enabled the synthesis of linear water soluble poly(sorbitol adipate) and poly(sorbitol succinate) without the need to use high temperature and protection-deprotection chemistry which are often barriers to commercialisation. Our synthesis of poly(sorbitol adipate) using K_2_CO_3_ under supercritical conditions (synthetic and as an extraction medium) showed a selectivity of about 68% towards the primary hydroxyl groups; close to that obtained with Novozym 435 (80% selectivity). This promising selectivity when using K_2_CO_3_ was unexpected and the consistently higher molecular weights of the polyesters (compared to those obtained using the enzyme) allow synthesis of polyesters similar to those frequently used in surfactant design. Our approach ensured the targeted synthesis of linear poly(sorbitol adipate) and poly(sorbitol succinate) by selectively reacting the d-sorbitol as a diol at the more acidic sorbitol terminal primary hydroxyls, which are also less sterically hindered. This retention of linearity ensured that the polyesters were rich in pendent secondary hydroxyl groups and were consequently water soluble. The water-soluble polyesters were further derivatised using hydrophobic lauric and stearic acids giving rise to competitive surface-active properties with very promising reductions in surface tension (as low as 26 mN m^−1^) compared to a range of commercial petrochemical and bio-based surfactants currently on the market. CMC values were identified to be dependent on the hydrophilic : hydrophobic ratio of the surfactants, ranging from 0.003 to 0.5 wt%.

Poly(sorbitol adipate) stearate efficiently dispersed the hydrophobic dye coumarin 6 in water, almost doubling the loading of this compound compared to the commercial Tween™ 20. These polyol-polyester surfactants were all identified to aggregate with hydrodynamic sizes of 130–225 nm. Poly(sorbitol adipate) based surfactants displayed sizes ranging from 130–170 nm, and could therefore also find use in biomedical applications, where nanoparticles <200 nm are desired. Additionally, end-capping of the polyol-polyesters can improve the hydrolytic stability of the compounds and could improve stability compared to the polyglycerol succinate laurate surfactants currently on the market. Our use of scCO_2_ as a reaction medium and extractant also showed promise in synthesising higher MW chains doubling the MW achieved compared to conventional low pressure sparging. This is likely to be attributed to the reduction in product viscosity and facile water removal.

In conclusion, a cost-effective catalyst system and straightforward synthesis route towards linear and hydrophilic polyol-polyesters makes them interesting candidates for the next generation of bio-based surfactants.

## Conflicts of interest

There are no conflicts of interest to declare.

## Supplementary Material

PY-012-D1PY00415H-s001
