# Supplementary material for: Synthesis of water-soluble surfactants using catalysed condensation polymerisation in green reaction media
Source: Polym Chem. 2021 Apr 26;12(20):2992–3003. doi: 10.1039/d1py00415h (PMC8145305; doi:10.1039/d1py00415h)
Supplement: PY-012-D1PY00415H-s001 [file PY-012-D1PY00415H-s001.pdf]

Electronic Supplementary Material (ESI) for Polymer Chemistry.
This journal is © The Royal Society of Chemistry 2021

Support Information

Synthesis of Water-Soluble Surfactants Using Catalysed Condensation Polymerisation in Green Reaction Media

Amy R. Goddard[ab], Edward A. Apebende[b], Joachim C. Lentz[b], Kim Carmichael[a], Vincenzo Taresco[b], Derek J. Irvine[c &] Steven M. Howdle[b]

[a] *Croda Europe Ltd, Foundry Lane, Ditton, Widnes, WA8 8UB, UK.*

[b] *University of Nottingham, School of Chemistry, University Park, Nottingham, NG7 2RD, UK.*

c Centre for Additive Manufacturing, Faculty of Engineering, University of Nottingham, Nottingham, NG7 2RD, UK

Experimental section

Materials and analysis

D-sorbitol (≥ 98%), adipic acid (≥ 99%), and succinic acid (≥ 99%), were purchased from Sigma Aldrich (UK). All reagents were dried *in vacuo* prior to use (20 ^{o}C, 100 mbar). *Para*-toluene sulfonic acid (*p*TSA), and potassium carbonate (K_2CO_3) were purchased from Fisher Scientific (UK) and Sigma Aldrich (UK) and used as received. Novozym 435 was kindly donated to us by Novozymes (Denmark), consisting of *Candida Antarctica* Lipase B (CalB) immobilised on cross-linked acrylic resin beads (10 wt%), stored at 2-8 °C and dried *in vacuo* (RT, 100 mbar) before use. All solvents were analytical grade and used as received. Carbon dioxide (minimum purity 99.9%) was purchased from BOC gases (UK) and used as received.

Nuclear magnetic resonance: Conversion of each reaction and degree of polymerisation were determined using ^{1}H nuclear magnetic resonance. Samples were dissolved in $CDCl_3$ and analysed using a Bruker DPX 400 MHz spectrometer operating at 400 MHz (^{1}H) and 100 MHz (^{13}C). Chemical shifts were assigned in parts per million (ppm). All spectra were obtained at ambient temperature (22 °C 1). MestReNova 6.0.2 copyright 2009 (Mestrelab Research S. L.) was used for analysing the spectra.

Potentiometric Titrations were carried out following standards commonly employed in industry for the determination of acid value (AV) and hydroxyl value (OHV) of polyesters. These titrations are used to gain information on the number of end-groups, molecular structure and the molecular weight of the polymers. The titrations were carried out in a Metrohm 785 DMP Titrino autotitrator (Metrohm, UK). Dynamic titrant addition was employed, and the equivalence point was determined using a programmed disperser, pH probe and the software tiamo 2.0. Standardised titrants were always used and all measurements were made in duplicate. Acid values (AV) were determined as follows:
Known quantities of polyester (0.25-0.5 g) were dissolved in 25-50 mL 50:50 (v/v) MeOH:H_2O to reach a concentration of 10 g/L. Solutions were titrated with aqueous NaOH (~ 0.1 M). AV titrations were performed at the start of the reaction (AV_{t0}) and at time t (AV_t).[1, 2]
The AV was used to determine reaction conversions ($PA^{\%}$) and predict the MW of the products according to the equations:

(I) $$AV = \frac{T \times M \times 56.1}{W}$$

(II) $$PA^{\%} = \frac{AV_{t0} - AV_t}{AV_{t0}} \times 100$$

(III) $$Mn^{AV} = \frac{56100}{AV_t}$$

Where T: volume NaOH (aq) titrated to reach the equivalence point (mL), M: molarity of NaOH (aq), W: weight of sample (g), AVx: acid value at the start (t0) and end (t) of the reaction. As acid values are defined by the number of mg KOH equivalent to the acid content of one gram of sample,[1] the mass of KOH (56.1 g/mol) has been used for calculations.

The hydroxyl value (OHV) of the polyesters was determined by hydroxyl end-group titrations using aqueous NaOH (~ 0.6 M). It was determined to ensure that the synthesised polyesters retain their hydrophilic OH groups and to assess the ratio between OH:COOH groups for each compound. The technique is based on reacting a compound with free hydroxyl groups with a known excess of acetylating reagent (a solution of acetic anhydride in pyridine 5% (V/V)). Followed by hydrolysing the unreacted acetic anhydride to acetic acid by treating with deionized water and then titrating the resulting solution with NaOH. A typical OHV determination was performed as follows:

0.25 g of polyester were accurately weighed into a 250 mL RBF. Acetylating reagent was added to the RBF (5 mL) and a water condenser was attached to the flask neck. The solution was heated (to ~ 90-100 °C) using a heating block for 1 hour and stirred magnetically to allow the free hydroxyl groups to react with the acetic anhydride. After 1 hour; 10 mL deionised water was added through the top of the condenser and the mixture was heated for a further 10 minutes to hydrolyse the remaining unreacted acetic anhydride to acetic acid. The RBF was removed from the heating block and allowed to cool to RT. Once cool, the condenser was washed down with THF (90 mL). This solution was placed on the stirring mantel of the auto-titrator and titrated against a 0.6 M NaOH solution. The measurement was performed in duplicate, along with a blank which followed the exact same procedure with the exception that the sample was omitted. The OHV and the degree of polymerizations were then calculated from the resulting data using the following formulae:

(IV) $$OHV_t = \frac{(B - T) \times M \times (56.1)}{W} + AV_t$$

(V) $$DP^{OHV} = \frac{OHV_t}{AV_t \times 4}$$

Where B: volume NaOH (aq) required for the blank (mL), T: volume NaOH (aq) required for the sample (mL), M: molarity of NaOH (aq), W: weight of sample (g), AV_t: acid value at the sample (mg KOH per g). As the hydroxyl value is defined by the number of mg KOH equivalent to one gram of sample,[1] the mass of KOH (56.1 g/mol) has been used for calculations. When determining DP^{OHV} the AV_t has been multiplied by 4 as in theory there will be 4 hydroxyl groups for each repeating *D*-sorbitol unit along the polymer backbone. **Table S1** below shows the data obtained from the potentiometric measurement of PSA with different catalysts.

Estimation of theoretical acid and hydroxyl values

A repeat unit of PSA has a molar mass of 287 g/mol. PSA of 1700 g/mol therefore has a degree of polymerization of 5.9. In an equimolar reaction of *D*-sorbitol and adipic acid, if the reaction would go to completion and only the primary alcohols react, there would be 4 free hydroxyl groups per repeat unit of PSA. We would also have one terminal hydroxyl and carboxylic acid group. That would be about 25 free hydroxyl groups and 1 free carboxyl group in the 1700 g/mol PSA example. The theoretical OH:COOH ratio for this polymer is therefore 25:1. **Table S1** shows that the acid value (AV_t) is 34 while the hydroxyl value (OHV_t) is 677 which lead to an experimental OH:COOH ratio of about 20:1. In the same way, the theoretical OH:COOH value of PSA of 1100 g/mol synthesized with Novozym is , the value for PSA of 3300 g/mol is estimated to be 45:1 while that of PSS of 5900 g/mol is 87:1.

Table S1 Comparing the potentiometric titration data for the polymerization of poly(sorbitol adipate) and poly(sorbitol succinate) using bio- and chemo-catalysts .

Entry	Polymer	Catalyst	Ratio [S]:[A]	AV_t (mg KOH/g)	$PA^{\%}$	OHV_t (mg KOH/g)	OH:COOH Ratio	DP^{OHV}	Mn^{AV} ($gmol^{-1}$)
1	PSA[b]	pTSA	2:1	35	84	n/a	n/a	n/a	1600
2	PSA[b]	N435	1:1	55	84	729	13:1	3.3	1100
3	PSA[b]	K_2CO_3	1:1	34	90	677	20:1	5.1	1700
4	PSA[a]	K_2CO_3	1:1	17	95	632	37:1	9.3	3300
5	PSS[a]	K_2CO_3	1:1	9	95	764	85:1	21.2	5900

[a]Polymerization was performed in $scCO_2$. [b]Polymerization was carried out in bulk. Note that the OH:COOH ratio of the polyesters is likely a slight underestimation compared to the true value, as acetylation of all hydroxyl groups would be challenging due to steric hindrance. This underestimation is seen in both the K_2CO_3 and the enzyme catalyzed samples.

Bulk polycondensation

Novozym 435 catalyst

D-sorbitol (5.00 g, 27.5 mmol) and adipic acid (4.02 g, 27.5 mmol) were transferred into a 50 mL three-neck round bottom flask (RBF) equipped with a mechanical stirrer and argon sparge. The reagents were heated (120 °C, approx. 15 min) under mechanical stirring (300 RPM) until molten. Reaction temperature was lowered (95 °C) and Novozym 435 (10 wt% relative to monomers) was added. The reaction was terminated after 48 hours by allowing the reaction mixture to cool to ambient temperature. Once cooled the product was dissolved in a 1:1 (v:v) mixture of MeOH and H_2O and filtered to remove the enzyme beads. The filtrate was recovered by solvent evaporation under reduced pressure and cooled (-18 °C) before being freeze-dried (Mini-Trap freeze dryer, LTE Scientific, UK).

Para-Toluenesulfonic acid catalyst

D-sorbitol (5.00 g, 27.5 mmol), adipic acid (2.01 g, 13.8 mmol), and pTSA (2 wt% relative to monomers) were added to a RBF. The mixture was heated (120 °C) and mechanically stirred (300 RPM). The reaction was terminated after 24 hours by decanting the reaction mixture into a ceramic crucible. The collected product was dried in vacuo (100 mbar, ambient temperature, overnight).

Potassium carbonate catalyst

D-sorbitol (5.00 g, 27.5 mmol), adipic acid (4.02 g 27.5 mmol), and potassium carbonate (5 wt% relative to monomers) were added to a RBF. The mixture was heated (120 ^oC) and mechanically stirred (300 RPM). The reaction was terminated after 48 hours by decanting the reaction mixture into a ceramic crucible. The collected product was dried in vacuo (100 mbar, ambient temperature, overnight).

Varying dicarboxylic acid chain length in base catalysed bulk polycondensation

D-sorbitol (20.00 g, 110 mmol), dicarboxylic acid (110 mmol, **Table S2**), and potassium carbonate (5 wt% relative to monomers) were transferred into a 100 mL three-neck RBF equipped with a mechanical stirrer and argon sparge. The mixture was heated (120 ^oC) and mechanically stirred (300 RPM). The reaction was terminated after 48 hours by decanting the reaction mixture into a ceramic crucible. The collected product was dried in vacuo (100 mbar, ambient temperature, overnight).

^{1}H-NMR (DMSO-d_6, 400 MHz) at 25 °C, δ = 5.05-3.31 (m, 8H, C***H***O and C$\boldsymbol{H_2}$O, *D*-sorbitol), using: adipic acid, δ = 2.35-2.25 (m, 2H, ROOCC$\boldsymbol{H_2}$), 2.23-2.18 (m, 2H, HOOCC$\boldsymbol{H_2}$) 1.58-1.48 (m, 4H, C$\boldsymbol{H_2}$*),* succinic acid: δ = 2.53-2.65 (m, 4H ROOCC$\boldsymbol{H_2}$), 2.43-2.37 (m, 2H C$\boldsymbol{H_2}$$CH_2$COOH), 2.29-2.22 (m, 2H CH_2C$\boldsymbol{H_2}$COOH).

Table S2 Masses of dicarboxylic acid used in bulk polycondensation.

Dicarboxylic acid	Mass (g)
Adipic acid	16.05
Succinic acid	12.99

Polycondensation of D-sorbitol and natural diacids in supercritical CO_2

Reactions were performed in a 60 mL high pressure autoclave described previously. In a typical reaction, D-sorbitol (5.00 g, 27.5 mmol), adipic acid (4.02 g, 27.5 mmol), and potassium carbonate (5 wt% relative to monomers) was added to the base of the autoclave. The autoclave was degassed using a flow of $scCO_2$ (2 bar) for 15 minutes before being sealed and pressurised (55 bar) by addition of $scCO_2$. The autoclave was subsequently heated (120 ^oC) before additional $scCO_2$ was added to raise the internal pressure to 240 bar. Consequently, the reaction was either allowed to proceed, or extracted during the last 15 minutes of every hour (6, 12, 24, 48 h). Products was collected by allowing the autoclave to cool to ambient conditions and depressurising slowly. The results of the synthesis for PSA and PSS are shown in **Table S3** below.

Table S3 Reaction of *D*-sorbitol and natural diacids in a 1:1 ratio using $scCO_2$ with extraction at 120 °C.

Entry	Reaction system	Polymer	Time (h)	Conv. % [a]	$PA^{\%}$ [b]	Mn^{AV} (g/mol) [b]
1	$scCO_2$	PSA	24	81	86	1500
2	$scCO_2$	PSA	48	91	95	3300
3	$scCO_2$	PSS	48	89	95	5900
4	Bulk	PSA	48	90	90	1700

[a] Determined by ^{1}H-NMR, [b] PA$^{\%}$ and Mn^{AV} have been determined by acid value titrations. Results from the polymerization of PSA in the bulk have been included for comparison. Note the difference in Mn^{AV} at 48 hours for PSA when using scCO_2 as a solvent and extractant compared to the same reaction in the bulk.

End-capping of sorbitol-based polyesters with lauric and stearic acid

Reagents (**Table S 2**) and catalyst (K_2CO_3, 5 wt% w.r.t. fatty acid) were transferred into a 50 mL 3 neck RBF equipped with a mechanical stirrer. The mixture was degassed (15 min, argon) before being immersed in a pre-heated oil bath (120 ^{o}C) and allowed to react (24 h, 300 RPM, argon sparged). Following the reaction, the product was decanted and allowed to solidify before washing (chloroform, approx. 15 mL). The collected product was dried in vacuo overnight prior to analysis.

Table S4 Reagents table for the end-capping of sorbitol-based polyesters. PSS (poly sorbitol succinate, 5900 Da), PSA (poly sorbitol adipate, 1700 Da).

Entry	Polymer	Mass (g)	mmol	Fatty acid	Mass (g)	mmol
1	PSA	5	2.9	Lauric	0.75	3.7
2	PSA	5	2.9	Stearic	1.00	3.5
3	PSS	10	1.7	Lauric	0.50	2.5
4	PSS	10	1.7	Stearic	0.75	2.6

Surfactant performance testing

Tensiometry

Surface tension (σ) measurements were made using the du Noüy ring method and a bubble tensiometer, measuring equilibrium and dynamic surface tensions, respectively. Equilibrium surface tension measurements were made on a Lauda Tensiometer TD3 using water (60 mL) at a polyester concentration of 0.5% w/v while bubble tensiometer measurements were made on a SITA t100 Bubble Pressure tensiometer with aqueous solutions containing 1% wt/v of polyester. The polyesters, poly(sorbitol adipate) and poly(sorbitol succinate) along with the corresponding laurate and stearate derivatives were all analysed by both methods. The following commercial surfactants; Tween™ 20, Tween™ 28, Tween™ 80, Pluronic™ L35, Pluronic™ L121 and NatraGem™ E145 were also assessed for comparison. All measurements were performed at room temperature (20 °C) and analysis was automated, with repeats made until a standard deviation of ≤ 0.1 mN/m was obtained (≥ 5 measurements). Adapted from Zuidema and Waters.[3]

Critical micelle concentration determination

The CMC was determined tensiometrically using an automated Wilhelmy plate. The system contains a fully automated micro dispensing unit which enables a high number of measuring points at a broad concentration range (0.1-10000 mg/L).

Dynamic Light Scattering (DLS) and Z-potential

Particle size analyses were performed by DLS utilizing a Zeta sizer Nano spectrometer (Malvern Instruments Ltd) equipped with a 633 nm laser at a fixed angle of 173°. The same instrument was used to measure the Z-potential of the produced NPs. Analysis was performed at 25 °C on a 1 mL sample (at a concentration ranging from 0.06 - 0.50 wt%). All experiments were performed in triplicate on the same sample.

Loading of micelles

Polysorbitol based surfactants (0.1 g) were added to water (1 mL) in order to make a 1 w/v% stock solution. Coumarin 6 (0.1 mL, 1 w/v% in DCM) was added to the stock solutions. DCM was evaporated and the solutions were agitated overnight. The samples were filtered (0.45 m, Millex. L.G, Millipore, USA) and diluted 6-fold in THF before being analysed by UV-Vis spectroscopy.

Dispersion of Hydrophobic dye

Furthermore, all of the solutions were passed through 0.45 µm syringe filters to eliminate undissolved coumarin 6, so it is possible that larger self-assembled compounds have been removed during this step. This could be a concern for surfactants which pack into larger aggregates.

^{1}H-NMR of PSA

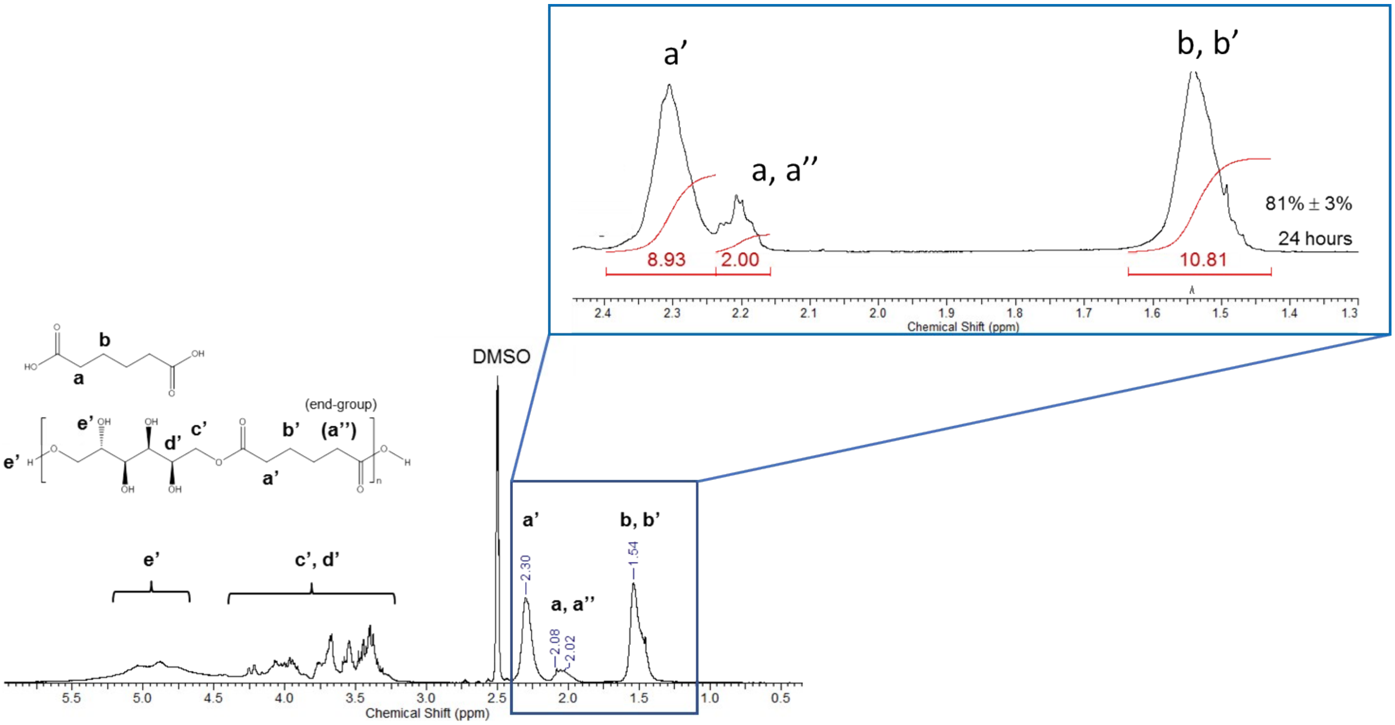

Figure S1 Typical ^{1}H-NMR spectrum of poly(sorbitol adipate), synthesised using *D*-sorbitol and adipic acid (81% conversion), analysed using DMSO-d$_6$. Note the overlap in signals from HOOCC*H$_2$*- in the monomer and polymer end group. **Inset.** Integrated values of monomeric adipic acid (ROCCH2-) to its reacted (oligomer or polymer).

Mass spectrometry of products

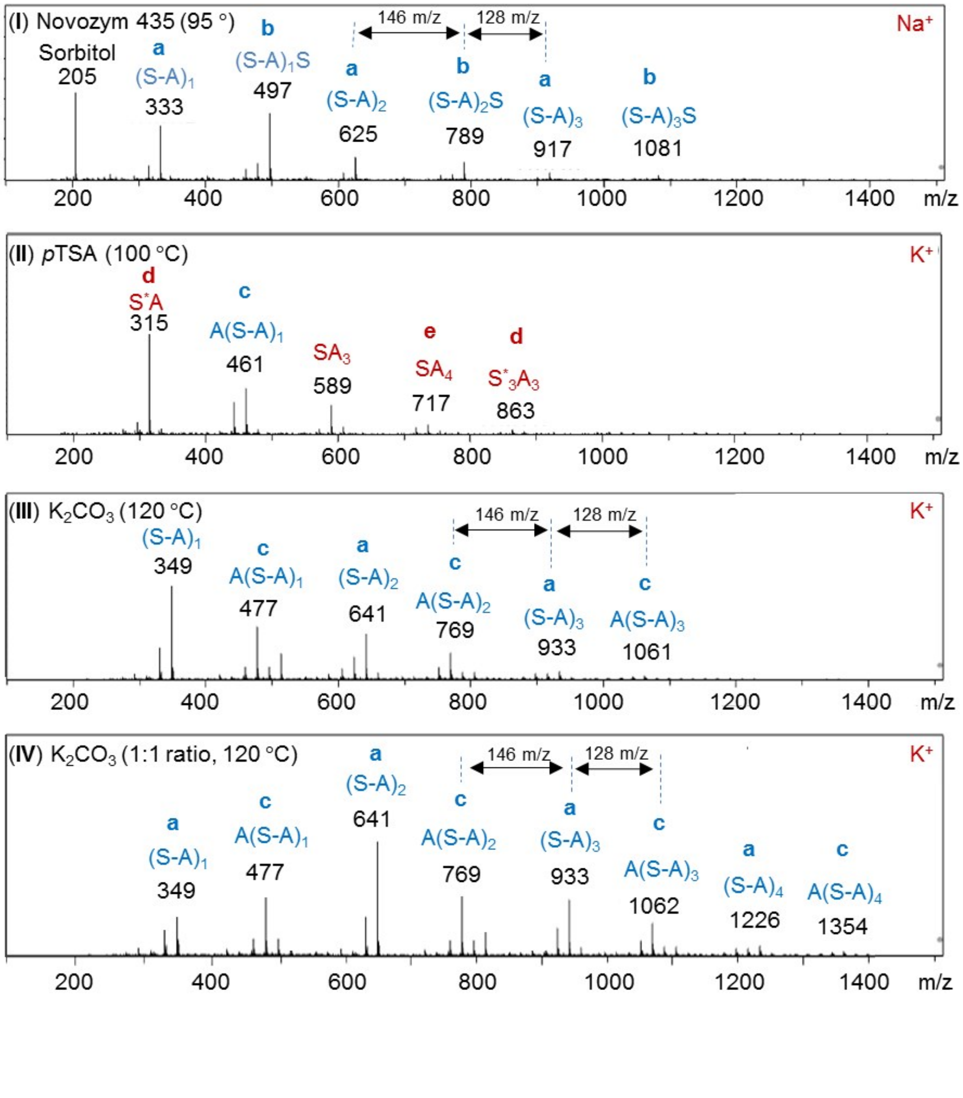

Figure S2 ESI-ToF MS spectra of poly(sorbitol adipate) synthesised using a 2:1 molar ratio of *D*-sorbitol (S) to adipic acid (A), catalysed by (I) Novozym 435 (95 °C), (II) *p*TSA (100 °C) and (III) K_2CO_3 (120 °C). Spectrum (IV) shows poly(sorbitol adipate) synthesised using a 1:1 molar ratio of reagents and K_2CO_3, included as a comparison. Note the very strong similarities between the spectra of the polymers synthesised enzymatically and using K_2CO_3, and the increase in higher MW peaks when using equimolar amounts of reagents. The corresponding ion adduct (Na^+ or K^+) is displayed in the right-hand corner of each spectrum. S^* = sorbitan.

Quantitative ^{13}C-NMR of PSA

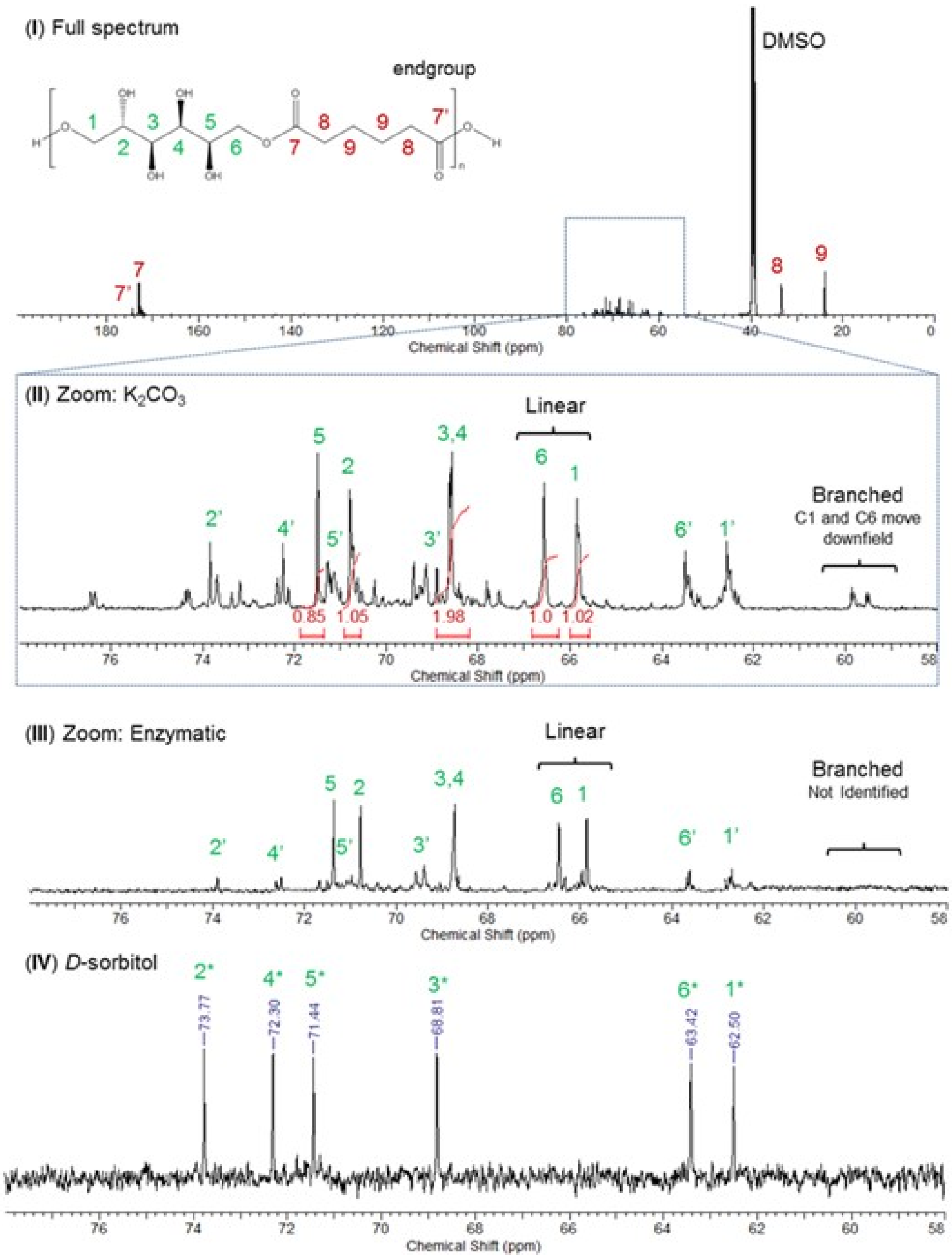

Figure S3 Quantitative ^{13}C-NMR spectra in DMSO-d_6 of poly(sorbitol adipate), synthesised at a 1:1 molar ratio of reagents using (I) K_2CO_3, with (II) highlighting the area of the spectrum associated with the polyol repeat unit. As a comparison (III) shows the equivalent compound synthesised enzymatically and (IV) shows *D*-sorbitol (not quantitative). Note the similarity between products synthesised using K_2CO_3 and Novozym 435, especially peaks at ~ 66 ppm highlighting derivatisation at the primary hydroxyl positions on *D*-sorbitol.

Solubility testing in $scCO_2$

Solubility tests in $scCO_2$ were carried out in a high-pressure fixed volume view cell. This was used to determine the temperature at which the reactants would liquefy or solubilise, as in experiments performed by Bratton *et al*.[4]

The melt temperature of the reagents (95 °C and 150 °C for *D*-sorbitol and adipic acid respectively, at atmospheric pressure) was not visibly affected by the addition of $scCO_2$ when heated up to 100 °C as shown in the view cell below. This is highly beneficial when conducting $scCO_2$ extraction. Lack of solubility in $scCO_2$ ensures the reagents and product remain in reaction, whilst the $scCO_2$ soluble condensate is removed.

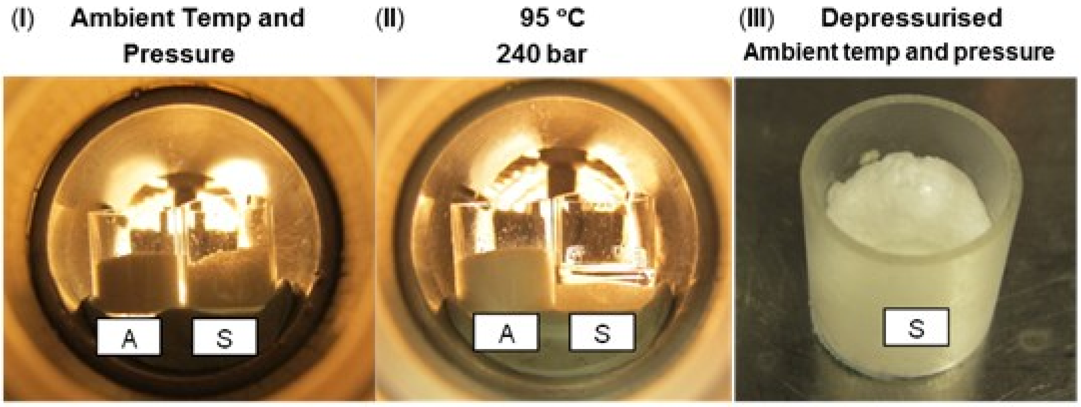

Figure S4 Transitions observed for adipic acid (A) and D-sorbitol (S) in the view cell at **(I)** RT and ambient pressure, where the reagents are white powders and **(II)** at 95 °C and 240 bar where D-sorbitol starts to liquefy (which is the same T_m as at ambient pressure), while adipic acid remains a solid. **(III)** Shows the foaming of D-sorbitol post-depressurisation.

Upon depressurisation significant foaming was observed for both reagents, indicating that $scCO_2$ can penetrate the reagents **(Figure S 4, III)**. This could be an advantage of employing $scCO_2$ as it may lead to a reduction in viscosity, as identified previously when processing polyesters such as PCL and PLA.[5, 6]

^{1}H-NMR of PSA-L

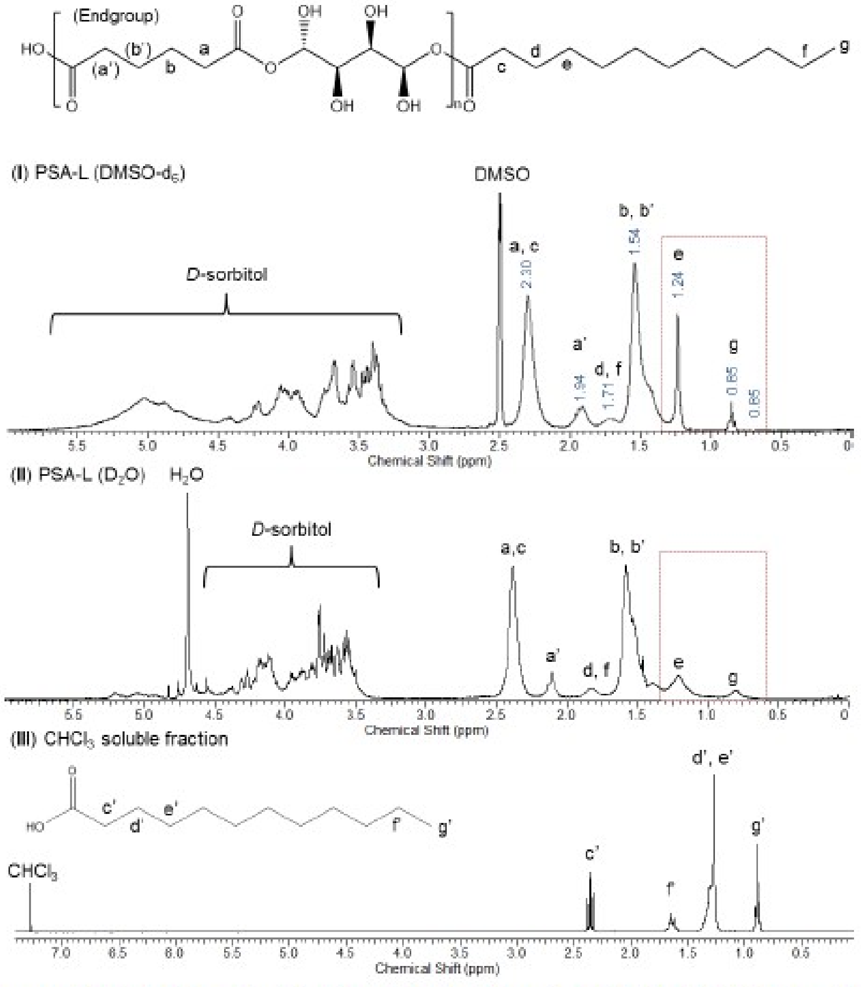

Figure S5 ^{1}H-NMR spectra of poly(sorbitol adipate) laurate (PSA-L), in **(I)** DMSO-d_6 and **(II)** All D_2O. Peaks are clearly detectable in DMSO, whilst the lauric acid resonances (red rectangles) are broadened and strongly suppressed in water. This strongly supports the idea that aggregates with a hydrophobic core are formed in aqueous environments. The fraction collected from purifying PSA-L after 6 hours using $CHCl_3$ has been included as **(III)** and is identified to be unreacted lauric acid.

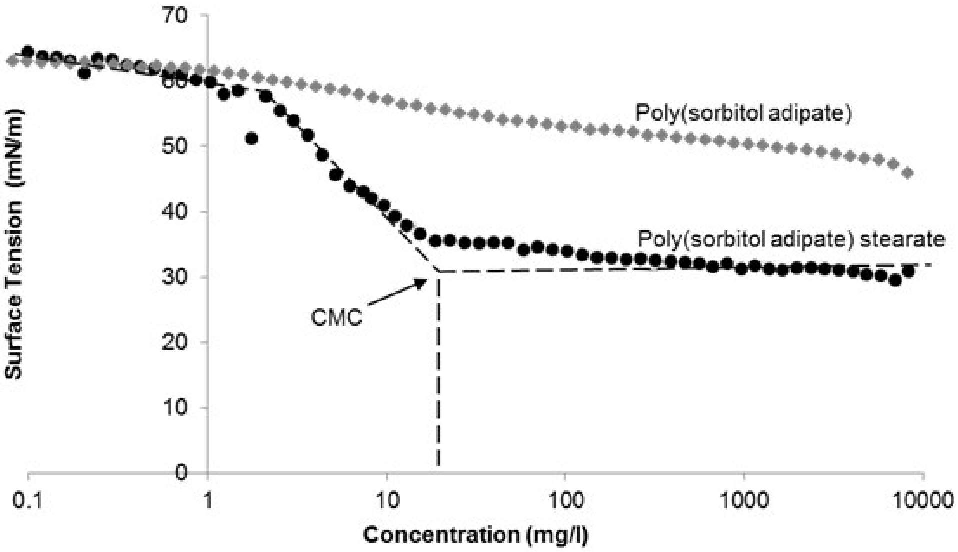

Figure S6 Surface tension measurements plotted against concentration for poly(sorbitol adipate) (♦) and poly(sorbitol adipate) stearate (●) (log scale). The extrapolation is included for clarity and is determined using the equipment software.

References

1. A. J. O'Lenick, *Surfactants: Strategic Personal Care Ingredients*, Allured Pub. Corp, Illinois, U.S.,, 2005.
2. X. Yin, H. Duan, X. Wang, L. Sun, W. Sun, H. Qi and L. Ma, *Progress in Organic Coatings*, 2014, **77**, 674-678.
3. H. Zuidema and G. Waters, *Industrial & Engineering Chemistry Analytical Edition*, 1941, **13**, 312-313.
4. D. Bratton, M. Brown and S. M. Howdle, *Macromolecules*, 2003, **36**, 5908-5911.
5. S. Curia and S. M. Howdle, *Polymer Chemistry*, 2016, **7**, 2130-2142.
6. C. A. Kelly, S. M. Howdle, K. M. Shakesheff, M. J. Jenkins and G. A. Leeke, *Journal of Polymer Science Part B: Polymer Physics*, 2012, **50**, 1383-1393.
